# The future of sickle cell disease therapeutics rests in genomics

**DOI:** 10.1242/dmm.049765

**Published:** 2023-02-23

**Authors:** Ambroise Wonkam

**Affiliations:** McKusick-Nathans Institute and Department of Genetic Medicine, Johns Hopkins University School of Medicine, Baltimore, MD 21205, USA

## Abstract

Sickle cell disease (SCD) is the most-common monogenic recessive disease in humans, annually affecting almost 300,000 newborns worldwide, 75% of whom live in Africa. Genomics research can accelerate the development of curative therapies for SCD in three ways. First, research should explore the missing heritability of foetal haemoglobin (HbF) – the strongest known modifier of SCD clinical expression – among highly genetically heterogenous and understudied African populations, to provide novel therapeutics targets for HbF induction. Second, SCD research should invest in RNA therapies, either by using microRNA to target the production of HbF proteins by binding to the transcription machinery in a cell, or by directly mediating production of HbF or adult haemoglobin through injection of messenger RNA. Third, investigators should aim to identify currently unknown genetic risk factors for SCD cardiovascular complications, which will address mortality, particularly in adults. Now is the time for global research programs to uncover genomic keys to unlock SCD therapeutics.

## Teaser

**Summary:** Exploring genomics in sickle cell disease could identify new therapeutic targets, and will further our understanding of genetic risk and treatment of associated cardiovascular complications, such as stroke or kidney disease.

## Introduction

**Fig. 1.**
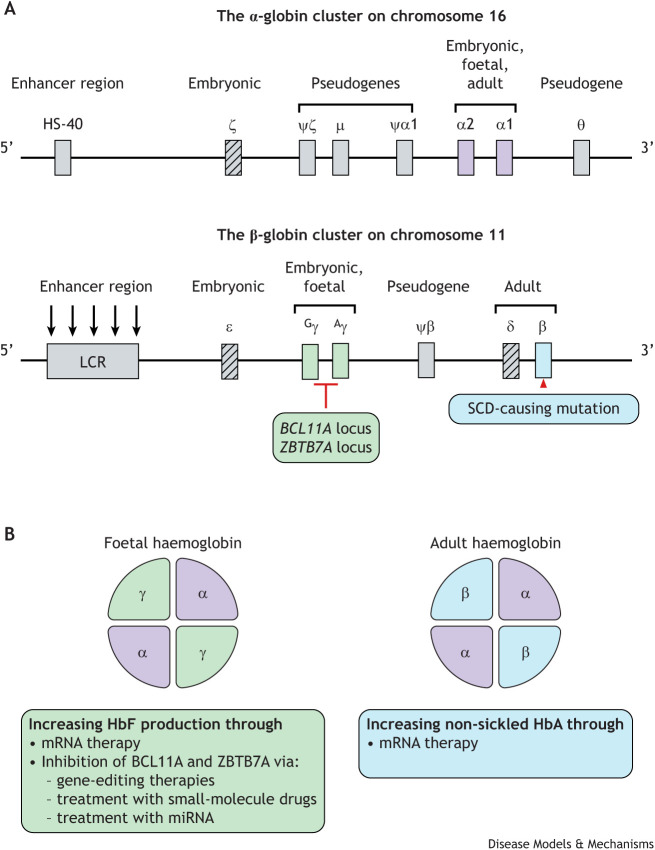
**Genomics-based therapeutic approaches targeting haemoglobin production.** During foetal life, foetal haemoglobin (HbF; also known as α_2_γ_2_) is the dominant form of haemoglobin. After birth, the level of HbF decreases progressively to ∼1% of its pre-birth level, when it is replaced by adult haemoglobin A (HbA; also known as α_2_β_2_). (A) Gene clusters of haemoglobin. Haemoglobin proteins are tetramers. The most common human HbA comprises subunits α2 and α1 (purple) encoded by genes within the α-globin gene cluster on chromosome 16 (top panel) and two beta (δ) subunits encoded by genes within the β-globin gene cluster on chromosome 11 (bottom panel). Human HbF also comprises the two α2 and α1 subunits encoded by genes on chromosome 16 but contains two gamma subunits Gγ and Aγ (green; encoded by genes *HBG2* and *HBG1*, respectively) (see Box 1) within the β-globin gene cluster on chromosome 11. Sickle cell disease (SCD) is caused by a single-nucleotide substitution within the beta-globin gene (*HBB*) on chromosome 11, yielding an abnormal β subunit. Haemoglobin production is tightly regulated by repressive transcription factors, such as BCL11A and ZBTB7A that specifically bind to the promoter of genes encoding HbF proteins. HS-40, enhancer element; LRC, locus control region. Grey boxes indicate pseudogenes and enhancer regions; grey striped boxes indicate less common embryonic (ζ, ε) or adult (δ) subunits. (B) Potential genomics-based therapeutic approaches to treat SCD can involve reactivation of HbF production through gene editing, or inhibition of repressive transcription factors through treatment with small-molecule drugs or miRNA (left). For therapeutics to increase production of either HbF or non-sickled HbA, it is possible to use RNA therapy (right) in an approach similar to the effective and successful development of the COVID-19 vaccine.

Sickle cell disease (SCD) is caused by a single nucleotide substitution in the beta-globin gene (haemoglobin subunit beta; *HBB*) (Fig. 1A), which encodes a component of haemoglobin (Hb), the protein complex that constitutes 70% of red blood cells (RBCs) and is responsible for transporting oxygen to all organs of the body. In SCD, the abnormal, sickled Hb (HbS) tends to polymerize in RBCs under specific conditions, such as dehydration, infection or lack of oxygen. This process causes RBCs to become deformed and rigid, and to take on a sickle- or banana-like shape. Sickled RBCs are most often destroyed in a process called haemolysis; they live an average of 20 days instead of 120 days, leading to anaemia. In addition, sickled RBCs tend to obstruct small blood vessels in all organs, leading to recurrent episodes of pain, and resulting in a lack of oxygen in critical organs, causing multiple organ damage. As a result, people living with SCD can suffer recurrent silent or overt strokes, and acute or chronic heart and kidney dysfunctions, leading to early mortality.

Owing to the partial protection conferred against malaria by the sickle mutation, SCD has become prevalent in areas of the world where malaria is endemic, particularly in Africa. It is estimated that, worldwide, ∼300,000 babies are born annually with SCD, with ∼75% of these births being in sub-Saharan Africa (Piel et al., 2013). In Africa, without appropriate treatment, at least 30% of children not treated for SCD die before the age of 5 years (Nnodu et al., 2021). The first clinical case of SCD was described in 1911 but, controversially, progress in drug development has been extremely slow, with only four United States Food and Drug Administration (FDA)-approved medications: hydroxycarbamide (also known as hydroxyurea, hereafter referred to as HU), L-glutamine, crizanlizumab and voxelotor. Of those, HU and voxelotor are both approved by the European Medicines Agency (EMA), but only HU is available in selected African settings. However, the impetus for developing novel therapies for SCD is supported by limited clinical acceptance of HU in certain areas of the world (Treadwell et al., 2022) owing to its potential side effects, including higher infections rates (Rankine-Mullings and Nevitt, 2022), as well as effects on fertility and reproduction, such as quantitative and qualitative semen and spermatogenesis abnormalities, although there is conflicting evidence of this (Berthaut et al., 2017; Joseph et al., 2021). To date, the only widely available curative approach to SCD is haematopoietic stem cell transplantation (HSCT), after which allogeneic stem cells that lack the genetic mutation yield healthy erythrocytes in the recipient. However, significant expertise is needed to render peri-transplant care for SCD patients, including blood transfusion support that might require extensive RBC antigen matching (Tanhehco et al., 2022). In general, paediatric SCD patients have better outcomes, whereas recovery of adults who – due to chronic inflammation and transplant-related toxicity – have acquired significant organ damage might be prolonged. Moreover, delayed immune reconstitution and high risk of infection or rejection remain significant problems, with SCD patients being at risk of death due to immunological responses. However, only a small proportion of SCD patients have matched sibling donors for HSCT. The urgency to discover novel SCD therapeutics has now been addressed by over 30 ongoing treatment intervention trials (Pace et al., 2021). As genetic factors influence many pathophysiological aspects of SCD, I propose here three main strategies to accelerate the development of novel curative therapies to treat SCD by using genomics research.

## Exploring the missing heritability of foetal haemoglobin in Africa to uncover therapeutic targets

During foetal life, foetal haemoglobin (HbF; also known as α_2_γ_2_) is the dominant form of Hb (Fig. 1), comprising subunits α_2_ and γ_2_. After birth, the level of HbF progressively decreases over 8-12 weeks to ∼1% of its pre-birth level and is replaced by adult haemoglobin A (HbA; also known as α_2_β_2_) (Fig. 1). Regulation of Hb production is tightly controlled by repressive transcription factors, such as BCL11A (Menzel et al., 2007) and ZBTB7A (Masuda et al., 2016) that preferentially bind to the promoter of the HbF genes *HBG1* and *HBG2* (Fig. 1A). Recent studies identified ZNF410, another transcription factor repressing expression of *HBG1/2*, which activates the expression of chromodomain-helicase-DNA-binding protein 4 (CHD4), a component of a repressor complex that is recruited to the gamma-globin (haemoglobin subunit gamma; *HBG*) promoter by BCL11A and ZBTB7A (Lan et al., 2021; Vinjamur et al., 2021). Genetic variations in HbF-modulating genes allow some individuals the capacity to continue producing HbF in adult life. Because of stressed erythropoiesis to compensate for the recurrent haemolysis and related anaemia, expression of HbF in most patients with SCD is higher compared to that in the general population. However, SCD patients with these genetic variants further retain the capacity to produce much higher levels of HbF (>8%) after birth and have fewer disease complications and longer life expectancy. This is because the presence of HbF in sickle RBCs delays polymerization of sickle-cell deoxyhaemoglobin (deoxy-HbS) and, thus, reduces clinical complications (Platt et al., 1994). Therefore, a main target for the development of new SCD therapy is the reactivation of HbF through gene-editing approaches (Box 1).

“[…] investigating HbF genomics at scale in African populations, with comprehensive functional analysis, will provide novel druggable targets for effective HbF induction […]”

**Box 1. Gene therapies for sickle cell disease (SCD)**

Two distinct approaches were used to obtain important recent advances in therapeutic/curative gene-editing curative for SCD. First, there is gene addition, which involves introducing a new gene into the patient’s genome to be integrated and expressed, e.g. targeting the mutated beta-globin gene (*HBB*) to boost production of non-sickled adult haemoglobin, or adding a gene with anti-sickling properties. The first gene therapy treatment of SCD was reported by Badat and Davies (2017), and introduced a modified β-globin gene (HbAT87Q) by using a lentiviral vector to prevent sickled haemoglobin (HbS) polymerization. After almost 20 months of follow up, median total haemoglobin levels increased in 35 patients, from 8.5 g/dl at baseline to ≥11 g/dl, and significantly reduced clinical manifestation of SCD (Kanter et al., 2022). Second, gene editing is another, potentially curative, therapy for SCD, aiming to modify the native gene itself. A highly successful gene-editing strategy for treating individuals who carry two mutant copies of *HBB* (resulting in HbS) and have either haemoglobin SS (HbSS) disease or HbS-beta-zero-(HSB 0) thalassemia, aims at targeting a transcriptional repressor, such as *BCL11A*, to reactivate foetal haemoglobin (HbF) – which does not sickle. This can be achieved with CRISPR-Cas9 disruption (Esrick et al., 2021; Frangoul et al., 2021), CRISPR-Cas12 mutation of enhancer sites within the promoters of *HBG1* and *HBG2* encoding haemoglobin subunits gamma 1 and gamma 2 (see Aγ and Gγ in Fig. 1A) (Métais et al., 2019; Traxler et al., 2016), and RNAi-induced suppression of *BCL11A* mRNA transcription by using short hairpin RNA (shRNA) expressed by a lentiviral-based vector (Esrick et al., 2021). Very promising results from a phase I study involving six patients with SCD showed robust and stable induction of HbF in all patients, as well as reduction or absence of clinical manifestations of SCD during the 6- to 29-month-long follow-up period (Esrick et al., 2021). Both gene addition and editing require collection and harvesting of haematopoietic stem cells from the SCD patient. Then, genetic modification ensues *in vitro*, followed by transplantation back into the patient after the residual marrow population has been ablated using chemotherapy, with major advantages of autologous haematopoietic stem cell transplantation (HSCT) over allogeneic HSCT. As we are still waiting for longer term follow-up data, it is too early to determine whether gene addition or gene editing is best. However, we definitively have more options when it comes to reactivating HbF.

**Fig. 2.**
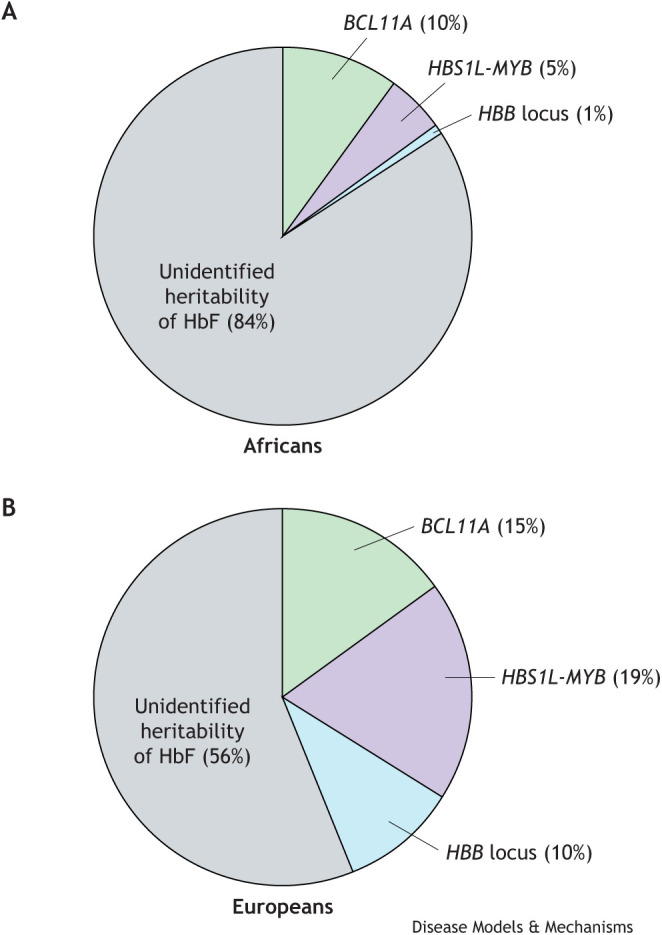
**Unidentified heritability of HbF.** (A,B) Differential proportion of HbF variance attributed to each locus among populations with African ancestry (Makani et al., 2017; Wonkam et al., 2014) (A) and populations with European ancestry (Menzel et al., 2007) (B), suggesting that new loci that are potential targets of HbF-manipulation therapies are more likely to be found among Africans. *BCL11A*, BCL11 transcription factor A; *HBB*, haemoglobin subunit beta; *HBS1L-MYB*, intergenic region between *HBS1L* and *MYB*.

However, it is estimated that >80% of gene variants accounting for heritability of enhanced HbF expression after birth are unknown in African populations (Fig. 2). The variants in the currently known HbF-modulating genes/loci, including *BCL11A*, the intergenic region between *HBS1L* and *MYB* (*HBS1L-MYB*), and the *HBB* locus, explain ≤16% of enhanced HbF expression after birth in African individuals with SCD (Makani et al., 2017; Wonkam et al., 2014). This compares with almost 50% of the variants leading to HbF persistence in adult Europeans being known (Menzel et al., 2007) (Fig. 2). This could mean that more variants in other HbF-controlling gene/loci are still to be discovered in African populations. Furthermore, the study discovering the most-recent known modulator of HbF – the repressive transcription factor *BCL11A –* had been performed using genome-wide association studies (GWAS) DNA arrays in populations of European ancestry, designed for this population (Menzel et al., 2007), meaning that these DNA arrays do not capture the high genetic diversity of understudied African populations (Martin et al., 2019). Indeed, there is evidence that variants identified in GWAS by using UK Biobank samples led to the development of polygenic risk scores associated with quantitative traits, such as blood indices, which then performed very poorly in populations with African ancestry (Martin et al., 2019). Moreover, very few GWAS have been performed in African populations, which, globally, make up only 2.5% of participants in currently available GWAS (Gurdasani et al., 2019). However, the limited African participants in GWAS account for almost 8% of all phenotype/disease associations in this study (Gurdasani et al., 2019). This high yield of data in the few GWAS that did include African populations is due to the high genetic diversity in African people – the oldest human population, and one that has accumulated over 300,000 years of human genome evolutionary history. Indeed, millions of genetic variants are either more common, rarer or specific to African populations, which also makes fine mapping of variants to disease/trait associations more productive. Therefore, expanding genomic research in populations of African ancestry, with appropriately designed GWAS arrays that capture the extent of genetic variation in that population, could uncover the missing heritability of HbF-promoting loci. Of note, the only GWAS performed for HbF levels in ∼1000 African individuals from Tanzania living with SCD did not uncover any new HbF-modifying loci (Mtatiro et al., 2014). This could suggest that additional modifier genes are rare and/or only have minimal effect. Alternatively, owing to the wide extent of unidentified variations in African populations, the GWAS DNA arrays were probably not suited to the genomic architecture of the Tanzanian population. I, therefore, propose that, to uncover new HbF-modifying loci, future studies only use specifically designed GWAS arrays developed from diverse African genome sequences, such as the one designed by the H3Africa Consortium. Already, this array is showing some promising results – i.e. the discovery of new loci associated with aberrant levels of low-density lipoprotein cholesterol – and the transferability of signals detected in the two large global studies consistently improved when the size of the African replication cohort was increased (Choudhury et al., 2022). The huge genetic diversity and the related complex haplotype structures in African populations are illustrated by the consistent discovery of millions of new variants (Choudhury et al., 2020; Retshabile et al., 2018; Sherman et al., 2019). These findings suggest that, to improve our understanding of HbF heritability and other complex traits, multicentric studies – that include thousands of individuals from major ethnolinguistic groups and diverse geographical regions from within Africa – are needed. Moreover, deep sequencing of suggestive loci should be performed systematically, followed or complemented by functional studies in cells and animal models, and extended to the innovative domain-focused CRISPR screen technology that has allowed identification of ZNF410 (Lan et al., 2021; Vinjamur et al., 2021) as well as of heme-regulated eIF2 alpha kinase (HRI), an erythroid-specific kinase that inhibits HbF translation in humans (Grevet et al., 2018). Ultimately, investigating HbF genomics at scale in African populations, with comprehensive functional analysis, will provide novel druggable targets for effective HbF induction – either through gene editing therapy or by using small molecules targeting those genes (Fig. 1B).

## Investigating the prospect of RNA therapy for SCD

Most RNA therapies can be sorted into one of three broad categories: those that target nucleic acids (either DNA or RNA), those that target proteins or those that encode proteins (Ying et al., 2008). In general, the use of RNA therapy has distinct advantages. First, most RNAs – i.e. microRNAs (miRNAs) or mRNAs – are naturally occurring molecules in human cells, with available mechanisms for their processing in place, as well as downstream target selection. Second, unlike gene editing/therapy, RNA does not interact with the recipient genome, a process associated with potential unwanted off-target integration, sometimes occurring in oncogenes.

For example, vector-induced leukaemia in response to enhancer-mediated mutagenesis occurred in 25% of patients in clinical trials for a rare genetic condition affecting the functions of white blood cells, called X-linked severe combined immunodeficiency (Hacein-Bey-Abina et al., 2003; Howe et al., 2008).

When it comes to short-term therapeutic potential for SCD, non-coding miRNAs – i.e. 18- to 25-nucleotide-long sequences that disrupt protein production by binding to the transcription machinery in a cell – are the most promising (Fig. 1B). Although much is still unknown about the mechanism of action of HU, there is consistent *in vitro* and *in vivo* evidence suggesting that HU induces HbF production via miRNAs (Mnika et al., 2019; Pule et al., 2016; Walker et al., 2011). Among adult patients with SCD living in Africa, we found that the majority of miRNAs that are differentially expressed in response to HU treatment are functionally associated with HbF-regulatory genes, including *BCL11A* (miR-148b-3p, miR-32-5p, miR-340-5p, miR-29c-3p) (Mnika et al., 2019). Response to HU and the subsequent miRNA expression are correlated with increasing HbF levels at baseline doses (for miR-494) and maximum tolerated doses (for miR-26b and miR-151-3p) of HU (Pule et al., 2016; Walker et al., 2011). Moreover, the use of miRNAs allows to target an entire pathway of HbF production, generating a stronger cumulative output compared with targeting a single gene. Research identifying more miRNAs that act on HbF production (Basak et al., 2020; Esrick et al., 2021) will provide an attractive new route for future SCD therapeutics that mimic HU-induced HbF production, while minimising potential consequences HU has on the whole cellular transcriptome, which could result in side effects (Berthaut et al., 2017). A second option of RNA therapy for SCD is to mediate direct production of HbF or non-sickled HbA through injection of exogenous mRNA. The latter functions similarly to some recently developed COVID-19 vaccines, has an outstanding safety profile and exceptional flexibility (Fig. 1B). One main challenge regarding this mRNA treatment is the delivery to target organs and cells; however, this is less of a problem in SCD because the bone marrow tissue generating RBCs is highly accessible. Moreover, blood is a renewable tissue, which allows an exit strategy if the mRNA therapy causes unpredictable and unwanted results. The delivery mode of this treatment could utilise lipid nanoparticles or polysaccharide-based nanoparticles that not only provide a shield but, also, harness existing cellular transport mechanisms to get the nanoparticle and its cargo into the bone marrow cell (Vissers et al., 2019).

Nonetheless, it is appropriate to highlight the potential challenges associated with RNA therapy as well as its delivery approaches. Results of therapeutic trials targeting non-coding RNAs (ncRNAs), such as miRNAs, have so far been inconclusive, with some studies reporting potent effects and others demonstrating limited efficacy or toxicity (Winkle et al., 2021). Regarding their delivery, it is known that most nanoparticles, preferentially, target liver cells (hepatocytes) and that most miRNAs have numerous target genes. Therefore, intravenous or intraosseous injection of nanoparticles encoding miRNAs that target *BCL11A*, *MYB* or other gamma-globin gene regulators probably have off-target effects affecting liver function and, possibly, other organ tissues. For example, studies in mice showed that miRNA therapy can be toxic, showing dose-dependent liver injury, ultimately, causing death in numerous experiments (Grimm et al., 2006). Therefore, for the purpose of future therapy, it is imperative to control intracellular RNA expression levels. Another challenge specific to SCD is that targeting *MYB* in haematopoietic stem cells and progenitors is likely to impair overall haematopoiesis, thereby affecting other blood cells beyond the production of RBCs.

“These examples of therapeutic prospects encourage future investment of resources and effort into the development of RNA treatment to benefit millions of patients living with SCD worldwide.”

Encouragingly, however, at least 11 RNA-based therapeutics are approved by the FDA and/or the EMA, aiming at gene modifications in liver, muscle or the central nervous system, and numerous RNA therapeutics are in phase II or III clinical development (Winkle et al., 2021). Moreover, packaging mRNA into an adenovirus vector is an efficient delivery approach (Lee et al., 2017). For example, in 2018, in both the United States and Europe, two RNA-based therapies were approved for hereditary transthyretin amyloidosis (Conceição, 2019), a progressive and, potentially, fatal disorder in which abnormal proteins build up within nerves and organs, such as the heart. More recently, Yang et al. described the successful suppression of abnormal bone formation in the ultra-rare genetic disorder fibrodysplasia ossificans progressiva, by using a combination of adeno-associated virus (AAV) gene delivery and miRNA silencing in a mice model (Yang et al., 2022). In addition, atypical effectors of RNA interference delivered by AAV can*, in vivo*, reduce the disease severity of retinitis pigmentosa caused by rhodopsin gene mutations (Orlans et al., 2021). These examples of therapeutic prospects encourage future investment of resources and effort into the development of RNA treatment to benefit millions of patients living with SCD worldwide.

## Developing genetic risk models to predict SCD complications

The implementation of screening of and comprehensive care for newborns has led to a drastic drop in SCD childhood mortality in the USA. However, mortality in adults has not changed over the past four decades in the USA because patients develop acute and chronic cardiovascular complications, such as stroke and kidney disease (Chaturvedi and DeBaun, 2016). The risk of these complications is also affected by genetic variation, such as variants in apolipoprotein L1 (*APOL1*) or deletions in alpha-globin genes (Geard et al., 2017; Sebastiani et al., 2005). Specifically, genetic coding variants in *APOL1* were evolutionarily selected in populations with African ancestry to confer resistance to trypanosome and prevent sleeping sickness (Cooper et al., 2017). Unfortunately, these variants, known as G1 and G2 alleles, are a frequent cause of the kidney disease APOL1 nephropathy (Genovese et al., 2010) in both patients with SCD (Adebayo et al., 2022; Basak et al., 2020; Geard et al., 2017) and in the general population with African ancestry (Ataga et al., 2022; Ekrikpo et al., 2020). Approximately 10-30% of African Americans and West/Central Africans carry two *APOL1* risk alleles (Ataga et al., 2022; Cooper et al., 2017; Ekrikpo et al., 2020). Interestingly, recent research has provided compelling data for novel therapeutic targets that may be useful for treating APOL1-nephropathies (Wu et al., 2021). Currently, antisense oligonucleotide drugs that inhibit APOL1 synthesis or function are in preclinical and clinical testing (Aghajan et al., 2019). Therefore, there is an urgent need to identify the full spectrum of genetic variants that modify clinical complications of SCD – including variants associated with long survivors, i.e. ≥40 years, living in unfavourable environments of Africa. For example, using exome sequencing, we have recently identified recurrent mutations in genes encoding components of the L-glutamine production pathways in such cohort (Wonkam et al., 2020). In addition, interactions of genes variants that have been evolutionarily selected and/or co-inherited with the SCD mutation need to be investigated (Esoh and Wonkam, 2021). Indeed, in this ‘long survivors’ group of SCD patients, we observed a high mutational burden in *CLCN6* and oxoglutarate dehydrogenase L (*OGHDL*). Previously, a rare exome variant in *CLCN6* – encoding a voltage-dependent chloride channel – has been associated with lower blood pressure (Yu et al., 2016). Given that increased blood pressure is a major risk factor for stroke in SCD (Cheng et al., 2014), the result suggests that SCD patients with some specific variants in *CLCN6* live longer due to a reduced risk of stroke. *OGHDL* is also important in arginine metabolism – a key factor in the haemolysis–endothelial dysfunction observed in SCD and a target for therapeutic interventions for SCD (Morris et al., 2013). The above and other, similar, results have the potential to be used as informative anticipatory guidance in clinical practice, while identifying targets of potential SCD therapeutics.

Gene–environment interactions must also be considered when developing novel therapeutics for SCD as, in Africa, important gene variants were evolutionarily selected due to endemic infectious diseases. Examples are a *G6PD* variant, or a 3.7-kb deletion in the alpha-globin gene causing alpha-thalassemia and being associated with malaria resistance and protection for kidney dysfunctions in SCD (Geard et al., 2017) or, as previously mentioned, variants in *APOL1* that are protective against *Trypanosoma* but increase susceptibility to kidney dysfunctions in SCD patients (Geard et al., 2017; Saraf et al., 2015). Because these variants, like the SCD mutation, are frequent in Africa, they are highly likely to have been co-inherited by numerous patients living with SCD (Esoh et al., 2021). The systematic evaluation of how their interactions influence the overall clinical severity of SCD is, therefore, needed. Moreover, a large inflammatory component is associated with SCD pathophysiology; this is emphasised in Africa, where high rates of bacteraemia and serious infections are most common in SCD patients (Williams et al., 2009). Thus, to investigate interactions between gene and environment are even more relevant for genetic associations between all individuals with SCD in Africa.

“Knowledge gained from these multicentre longitudinal multi-omics studies in Africa will allow the development of mathematical models to evaluate genetic risks, enabling SCD patient stratification in infants to cluster by severity, and to optimise treatment and care […]”

Understanding genetic variation in African populations has advanced greatly in the past few years, with multiple cross-ancestry studies of common diseases or health-related traits being performed in non-SCD patients, albeit being relevant to SCD patients. For example, in a large African ancestry cohort, the *APOL1* risk genotype and polygenic component of the genome-wide polygenic score has additive effects on the risk of chronic kidney disease (Khan et al., 2022). Another study found seven novel signals in UK Biobank data of African ancestry participants, including a cis-protein quantitative trait loci (cis-pQTL) for the gamma-glutamyl transferases gene group, as well as for *PIEZO1* and *G6PD* variants with impacts on Hb bound to sugar (Sun et al., 2022). All these studies should be expanded and employed in SCD patient cohorts, by using multicentre longitudinal studies and methodological approaches – including classic GWAS, whole-exome and whole-genome sequencing – together with multi-omics such as transcriptomics, metabolomics, proteomics and metagenomics. Knowledge gained from these multicentre longitudinal multi-omics studies in Africa will allow the development of mathematical models to evaluate genetic risks, enabling SCD patient stratification in infants by severity, and to optimise treatment and care accordingly.

## Perspectives

Why should we invest in expensive genetic studies while most countries in Africa, where SCD is most prevalent, are not even able to implement basic care, such as prophylactic penicillin? And how successful and equitable could such genomic research be, considering the technological challenges and high costs associated with currently available HSCT – the estimated cost of HSCT per patient ranging from $350,000 to $800,000, and gene-editing therapy as high as $1-$2 million (Leonard et al., 2020)?

Considering that the total cost of managing a patient living with SCD until the age of 50 exceeds $8 million (Leonard et al., 2020), the upfront high cost of HSCT or gene therapy/gene editing should be acceptable, and a concerted effort to explore new routes for therapeutics for SCD patients in all parts of the world is essential. This must include the development of an *in vivo* gene-therapy delivery system that bypasses the need for an autologous transplant and, possibly, makes worldwide application equitable. Moreover, investing in discovering novel therapeutic targets offers more options to reactivate HbF by using pharmacologic approaches, such as small molecule regulators that target HbF-modifying genes (Yu et al., 2020). As exemplified by the recent fast development and implementation of COVID-19 vaccines, I strongly believe such effort is possible and must, indeed, be made. Learning from recent failures in global vaccine distribution during the COVID-19 pandemic (Makoni, 2022), this effort should be accompanied by a mechanism to address the equity crisis by establishing centres of excellence for SCD care, particularly in Africa, and the help of international agencies, such as the World Health Organisation. This must be supported by concerted strategies from numerous stakeholders, including industry, national governments, SCD-patient-support groups, professional societies, international agencies and funding bodies with expanding mechanisms, such as the Cure Sickle Cell initiative launched in September 2018 by the National Heart Lung and Blood Institute, NIH, USA. Exploring genomics in SCD could also further our understanding of specific cardiovascular complications, such as stroke or kidney disease, that are both common in SCD and the general population. Moreover, investing in genomics with the view of developing new SCD treatments will provide a frame to develop treatments for other monogenic diseases.
